# Boosting people’s ability to detect microtargeted advertising

**DOI:** 10.1038/s41598-021-94796-z

**Published:** 2021-07-30

**Authors:** Philipp Lorenz-Spreen, Michael Geers, Thorsten Pachur, Ralph Hertwig, Stephan Lewandowsky, Stefan M. Herzog

**Affiliations:** 1 Center for Adaptive Rationality, Max Planck Institute for Human Development, Berlin, Germany; 2 School of Psychological Science and Cabot Institute, University of Bristol, Bristol, UK; 3 School of Psychological Science, University of Western Australia, Perth, Australia

**Keywords:** Psychology, Human behaviour

## Abstract

Online platforms’ data give advertisers the ability to “microtarget” recipients’ personal vulnerabilities by tailoring different messages for the same thing, such as a product or political candidate. One possible response is to raise awareness for and resilience against such manipulative strategies through psychological inoculation. Two online experiments (total $N= 828$) demonstrated that a short, simple intervention prompting participants to reflect on an attribute of their own personality—by completing a short personality questionnaire—boosted their ability to accurately identify ads that were targeted at them by up to 26 percentage points. Accuracy increased even without personalized feedback, but merely providing a description of the targeted personality dimension did not improve accuracy. We argue that such a “boosting approach,” which here aims to improve people’s competence to detect manipulative strategies themselves, should be part of a policy mix aiming to increase platforms’ transparency and user autonomy.

## Introduction

Online companies infer detailed information about people from the behavioural traces they leave on their platforms, giving advertisers the ability to exploit recipients’ personal characteristics and potential vulnerabilities (e.g., personality, political views or sexual preferences;^1,2^) by “microtargeting” them through messages that are specifically tailored to them^3,4^. Advertisers have always sought to maximize the match between their messages and presumed customers. Traditionally, they did so by using easily “observable” demographic features such as age and gender: There are few cosmetic ads in motorcycle magazines, and TV commercials rarely advertise toys at times when children are usually already in bed. Similarly, political parties and candidates have long been segmenting the electorate into blocks of voters in order to more efficiently allocate political ads^5^.

However, compared to traditional audience segmentation, “microtargeting” of individuals based on personal attributes can be ethically problematic for several reasons. First, it can exploit recipients’ inferred characteristics, including sensitive attributes such as sexual orientation, even without their knowledge or consent^4^. Second, tailoring messages for one and the same product or political candidate goes beyond persuasion and approaches manipulation, especially when the inferred vulnerabilities of recipients, such as specific anxieties, are being targeted^6^. In the political context, this can become particularly problematic, because it allows politicians to give, outside of the public’s sight, contradictory promises and pledges to different audiences^7^ while avoiding rebuttal by political opponents^8,9^.

Microtargeting can undermine transparency and autonomy, whenever targeted people do not know what data platforms hold, what can be inferred from those data, and how it is used to target them^10^—thus contributing to a growing knowledge gap between platforms and their users^11^. With increasing technological sophistication, these processes are becoming even more opaque for the public and for targeted individuals^12^.

Previous research conducted on Facebook has concluded that inferred personality dimensions can be used to enhance the effectiveness of ads: Participants were more likely to buy a product when they were targeted with an advertisement that matched their personality type (extravert or introvert;^3,13,14^). Other studies have found that personality-based targeting increased engagement, but did not consistently change attitudes towards a product^15^. In the political domain, personality-based matching of advertisement has also been found to be more effective in influencing political attitudes and voting intentions than non-matching advertising^16^.

Even though the persuasive effect of a single ad on a single individual may be relatively small^17^, the potential harms of political microtargeting scales up when employed widely^7^. Political online advertising, for example, generates billions of impressions on social media^18^, and it has been shown that even small visual details can affect voting intentions^19^. Facebook’s hidden ad-delivery mechanisms can increase biases^20^ and polarise political campaigns^8,9^. Last but not least, representative surveys across several countries found that opaque targeting practices—based on sensitive or protected attributes, like political views or sexual preferences—are at odds with public attitudes and that this disapproval holds across the political spectrum^21^.

Whatever the persuasive power of current practices, microtargeting lacks transparency and contributes to a growing knowledge gap between platforms, advertisers, and users. While platforms are becoming increasingly more sophisticated in collecting data and in customisation, there is a dearth of effective measures that could help counteract the adverse consequences of these developments.

One strategy to close the knowledge gap is to enhance users’ awareness of microtargeting practices. In light of fast and constantly changing targeting methods this approach may be more robust than attempts to regulate the platforms. It has been shown that advertisements are less effective when people find out that unacceptable practices (i.e., using information obtained from outside the platform or inferred without user input) have been used to target them^22^. However, current transparency measures, such as the “Why am I seeing this?” button on Facebook, provide only superficial information and have to be actively requested by users^23^. Thus, although platforms are required to disclose the data they hold about users, in practice, for most users this requirement fails to open the platforms’ “black box”. At present, the platforms’ transparency measures offer “nominal transparency”, with no real regard for whether people actually can easily access, read and gain insight into the information held about them and whether this transparency in name foster users’ autonomy. Aiming for *effective transparency*—which demonstrably enables users to understand what platforms do with their data and what users’ choices imply, and to then translate this knowledge into measurable behaviour—is an important step towards more acceptable business practices and towards regaining some autonomy for users (e.g., by prompting people to adjust their privacy settings;^24^).

Here, we pursue a cognitive approach inspired by research showing that people can be psychologically “inoculated” against misinformation^25^. For example, explaining misleading argumentation techniques reduces the influence of subsequently presented misinformation^26^. We report two experiments that test whether it is possible to inoculate people against personality-based microtargeting^3^. In all treatment conditions, we made participants reflect on the personality dimension being targeted—the extraversion–introversion spectrum—and examined whether this intervention would increase people’s ability to identify whether or not an advertisement is targeting them personally. Across experiments, we compared interventions that differed in their degree of specificity: (1) the most general intervention merely described the targeted personality dimension; (2) an intermediate intervention involved participants completing a short personality questionnaire (without providing feedback); (3) the most specific intervention provided participants with feedback on how they rank in their personality relative to others, based on their responses in the questionnaire.

If the success of the intervention depends primarily on people being aware of the personality dimension being targeted, then a general description may suffice to increase their sensitivity to being microtargeted. However, to the extent that people lack relevant self-knowledge^27,28^, or fail to spontaneously connect their self-knowledge with the advertisements, then more specific inoculation interventions may be necessary. We tested three inoculation interventions that are all instances of the class of “boosting” interventions, that is, interventions aimed at improving people’s competences to make better decisions in light of their own goals^29,30^.

Across two experiments, we test the following hypotheses, where hypotheses **H2a**, **H2b**, and **H2c** are mutually exclusive and assume that **H1** is supported:

- **H1**: A boosting intervention, which prompts people to reflect on and receive feedback about their relevant personality dimension, increases their ability to accurately identify ads that are targeted towards them.
- **H2a**: A boosting intervention increases people’s ability to accurately identify ads that are targeted towards them primarily by raising people’s awareness of the specific targeting strategy (i.e., the targeted personality dimension).
- **H2b**: A boosting intervention increases people’s ability to accurately identify ads that are targeted towards them only if people actively reflect on their own relevant personality dimension, while merely raising people’s awareness of the specific targeting strategy (i.e., the targeted personality dimension) is not sufficient to increase accuracy.
- **H2c**: Neither of the above mechanisms suffice; a boosting intervention only increases people’s ability to accurately identify ads that are targeted towards them if the intervention provides explicit feedback about people’s relative score on the targeted personality dimension (as in **H1**).

**Figure 1:**
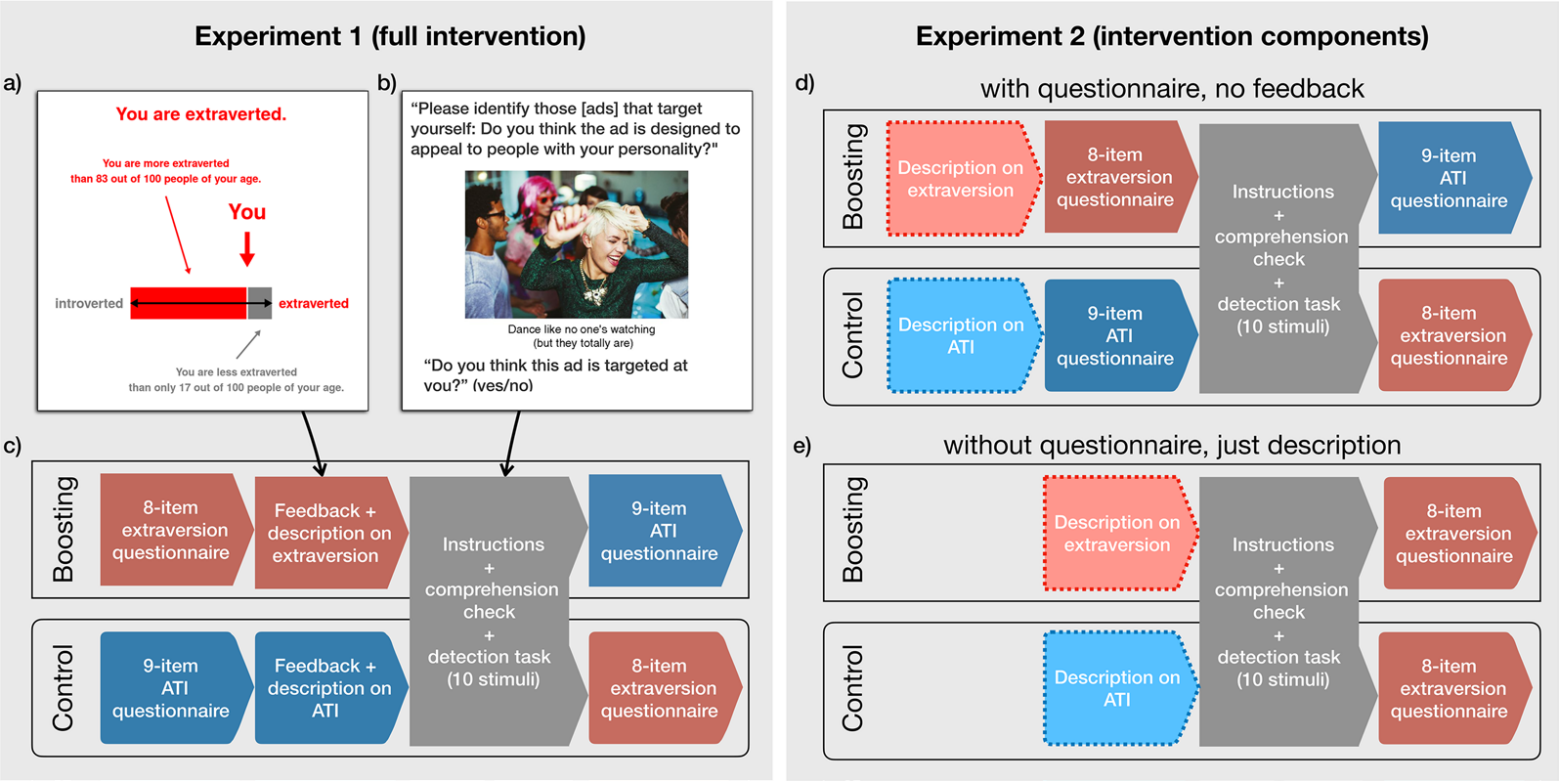
Description of Experiments 1 and 2. (**a**) Feedback screen shown to participants in Experiment 1 after they completed an 8-item personality questionnaire assessing their extraversion level (boosting condition), which includes feedback on their relative rank within an age-matched norm population (from^31^). (**b**) Experiments 1 and 2: Instructions of the detection task and example stimulus, taken from^3^ (for the full set of stimuli, see Table S1). (**c**) Design of Experiment 1, participants in the boosting condition received feedback about the relevant personality dimension (extraversion) before the detection task, whereas participants in the control condition received feedback about an irrelevant personality dimension (Affinity for Technology Interaction, ATI). That is, the only difference between the two conditions is that the order of the two personality questionnaires (plus the corresponding feedback) were swapped (i.e., before vs. after the detection task). (**d**) In Experiment 2, participants were only exposed to components of the full intervention used in Experiment 1: Participants did not receive any feedback before the detection task (“with questionnaire”) and (**e**) half of participants only read the description of either the relevant or irrelevant personality dimension (“without questionnaire”).

## Experiment 1

### Method

The preregistration of the study can be accessed at https://aspredicted.org/ez6h2.pdf and includes, among other things, the research question, hypothesis **H1**, the primary outcome variable, planned sample size, exclusion criteria, and the exact specification of the mixed-level logistic regression model used to analyse this experiment. We report all data exclusions, all manipulations, and all measures used in the study (see^32^). The experiment was programmed using *formr* (https://formr.org)^33^. All data and code are publicly available at https://osf.io/ne4r9/.

#### Materials and procedure

In the boosting condition, participants completed an 8-item extraversion questionnaire (see Supplemental Information Fig. S3). Based on their responses, they received personalized feedback (see Fig. 1a and Fig. S5) on their extraversion score relative to a large sample of online participants (from^31^ see Supplemental Information section 1 for more details); this was truthful feedback, calculated for each participant on the fly. Specifically, participants were told whether their personality tended more towards extraversion (“You are extraverted”) or introversion (“You are introverted”). A participant’s percentile was shown both numerically and visually, expressed as how many of 100 random people were more and less extraverted (for participants categorised as extraverts) or introverted (for participants categorised as introverts) than the participant themselves. The feedback was accompanied by a simple definition of extraversion adopted from Wikipedia (https://web.archive.org/web/20190801042657/https://en.wikipedia.org/wiki/Extraversion_and_introversion, see also Fig. 1a and S3). We enforced a 1-minute wait on the feedback screen to ensure that participants processed the feedback. The control condition followed the same procedure, but participants completed an unrelated, 9-item questionnaire tapping their propensity to naturally interact with technical systems (Affinity for Technology Interaction, ATI;^34^ for full questionnaire, see Fig. S4). The ATI feedback and the description of the dimension was presented in a format analogous to that used in the boosting condition (see Fig. S7).

We then presented 10 ads for beauty products (taken from Matz et al.^3^) in random order to the female participants, who were mirroring the population targeted in^3^. Five of the ads were specifically designed to target extraverts; five target introverts (for the full set of stimuli, see Table S1). Each ad consisted of a picture and a slogan. “Extraverted” ads emphasised socially stimulating contexts (e.g., “Love the spotlight”), whereas “introverted” ads emphasized socially less stimulating contexts (e.g., “Beauty isn’t always about being on show”). The original study^3^ validated the stimuli by showing that extraverted ads were rated as more extraverted than introverted ads (and vice versa).

Right before the beginning of the ad targeting detection task, participants received the following instructions: “In the following you will be shown ads that are all designed for women, but are additionally targeted at different personality types. Please identify those that target yourself: Do you think the ad is designed to appeal to people with your personality? Or do you think it is designed to appeal to people with a different personality?” That is, in this study, microtargeting was defined as addressing participants by tailoring ads to aspects of their personality. This was followed by a comprehension check (see Fig. S9): “Please complete the following sentence. For the following ads, I need to rate whether I think the ad is ...,” followed by the options “copied from a previous ad,” “targeted towards my personality type,” “appealing to me,” and “going to be effective when aired.” If participants did not select “targeted towards my personality type,” the question was repeated (max. two times) with the response options presented in a different order. As per preregistration, we included participants in the analysis only if they passed the comprehension check within a maximum of three attempts. For each ad, participants were then asked whether it was targeted towards their personality type: “Do you think this ad is targeted at you?” (“yes” vs. “no”; see Fig. 1b). Participants also indicated their decision confidence by responding to the question “How confident are you with your choice?” (Likert scale ranging from 1 = “not confident” to 5 = “very confident”).

To summarise the procedure: Participants were randomly assigned to one of two conditions. In the boosting condition, participants first completed the extraversion questionnaire and received feedback on their relative extraversion score (see Fig. 1a), then evaluated the targeting of the ads, and finally completed the ATI questionnaire and were given feedback on their relative ATI score (see Fig. S6). In the control condition, the position of the extraversion and ATI questionnaires (plus their respective feedback) was switched. Participants were asked to indicate their age in both the extraversion and the ATI questionnaire; this measure was used as a response consistency measure (see exclusion criteria). The study concluded with a question about education.

#### Participants

We collected responses from 318 participants (boosting condition $N=158$, control condition $N=160$, randomly allocated on the fly) via Prolific Academic, an online survey platform whose participants are more diverse and less familiar with experimental procedures than Amazon Mechanical Turk workers^35^. Mirroring the population targeted in^3^, we recruited female participants between the ages of 18 and 40 years who were UK residents fluent in English; we did not invite participants who already participated in a pilot study, via the prescreening functionality of Prolific. All participants provided their informed consent and received £2 for completing the study. No data that allows any identification of participants is reported here, all participants provided informed consent on the data handling.

Consistent with the preregistered exclusion criteria, we excluded participants for the following reasons: 25 participants for non-completion (13 in the boosting condition, 12 in the control condition), 2 participants for giving different responses to the two age questions (1 in the boosting condition, 1 in the control condition), 6 participants for failing the comprehension check (4 in the boosting condition, 2 in the control condition), 1 participant (from the boosting condition) with a relative extraversion percentile of exactly 50%, as no extraversion personality type can be assigned for participants with this value. The final sample thus comprised 284 participants, $N=139$ in the control condition and $N=145$ in the boosting condition. The median age of participants was 30 years (first and third quartile: $Q_1 = 26$ and $Q_3 = 34$ years).

#### Analysis

The primary dependent variable was a participant’s decision about whether or not a particular ad was targeted towards her personality (“yes” vs. “no”). We classified each participant as either extravert (percentile > 50%) or introvert (percentile < 50%) on the basis of their percentile rank for extraversion. Based on this categorisation, each participant’s decisions were then scored as either correct or incorrect. Specifically, a decision was scored as correct if an extraverted participant responded that an extraverted ad was targeted at her or an introverted ad was not targeted at her. A decision was scored as incorrect if she responded that an extraverted ad was not targeted at her or that an introverted ad was targeted at her. The opposite coding was used for introverted participants. For analysing the results, we used a Bayesian mixed-level logistic regression^36,37^ (for more details about the implementation see the Supplemental Information).

### Results

**Figure 2:**
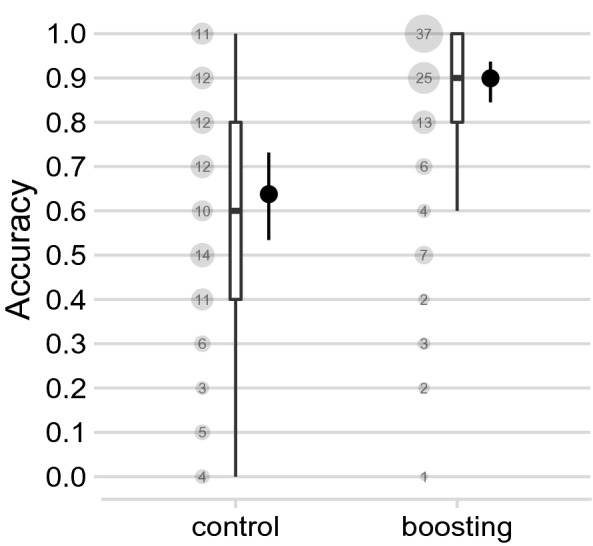
Effect of boosting and control interventions on the accuracy of detecting targeted advertisements (Experiment 1); see Fig. 1 for the experimental setup, where participants in the boosting condition received feedback about their extraversion prior to the task. Point ranges show the Bayesian point estimate and 95% Bayesian credible interval for the probability of correctly detecting a targeted advertisement (based on a Bayesian mixed-level logistic regression model; see Supplemental Information for details). In the boxplots, the box shows the the first, second (median), and third quartiles (the 25th, 50th, and 75th percentiles). The lower and upper whiskers extend from the respective end of the box to the largest value no further than 1.5 $\times$ IQR from the box (where IQR is the inter-quartile range, or distance between the first and third quartiles); outliers are not displayed. The area of the dots and their numbers denote the within-condition percentage of participants for each of the 11 possible values for a participant’s proportion of correct decisions (given the 10 ads). Figure produced using R version 4.1.0^38^.

Figure 2 shows that the results supported hypothesis **H1**: Relative to the control condition, participants in the boosting condition correctly identified, on average, 26 percentage points more ads targeted at them (95% Bayesian credible interval, CI 18–35)—raising the mean accuracy from 64% (95% CI 53–73) to 90% (95% CI 85–94). This difference corresponds to an effect size, expressed in terms of the “common language effect size”^39^, of $CL = 0.78$ (95% CI .70–.84), which here indicates the probability that a randomly selected participant from the boosting condition has a higher detection accuracy than a randomly selected participant from the control condition. A value of 0.5 would imply no difference and 1 would imply perfect separation between conditions. Additional analyses, detailed in the Supplemental Information (Figs. S10–S12), attest to the robustness of these results. To summarise, the intervention worked (a) for both extraverts and introverts, (b) different levels of education, (c) irrespective of whether participants were clearly or more tentatively classified as extravert or introvert; moreover, the effect (d) also emerged when we measured detection performance independently of any response tendency (lenient vs. strict), in terms of the area under the Receiver Operating Characteristics curve^40^ (AUC; based on participants’ confidence in their detection decisions) and (e) was stronger for extraverts than for introverts. The stronger effect for extraverts seems to come from the low baseline accuracy of moderately extraverted participants in the control condition who had a lot of room to improve in the boosting condition (Figs. S10 & S11); this phenomenon is asymmetrical and not observed for moderately introverted participants. Overall, these results demonstrate that it is possible to improve people’s ability to detect targeted advertisements through a short, simple boosting intervention.

## Experiment 2

Experiment 2 aimed to disentangle the components underlying the effects found in Experiment 1 by omitting individual parts of the intervention step-by-step and observing the resulting effects: Did the boosting intervention in Experiment 1 work because it (1) implicitly hinted at the targeting strategy of the advertiser by describing the relevant personality dimension (i.e., a mere description of the personality dimension suffices), (2) encouraged people to reflect on their own position on the relevant personality dimension by having them complete a questionnaire (without providing feedback), or (3) explicitly provided individual feedback on the relevant personality dimension (i.e., degree of extraversion vs. introversion)?

### Method

Experiment 2 was identical to Experiment 1, with the exceptions specified below. The preregistration can be accessed at https://aspredicted.org/a7k2g.pdf and includes, among other things, the research question, hypotheses **H2a**–**c**, the primary outcome variable, planned sample size, exclusion criteria, and the exact specification of the mixed-level logistic regression model used to analyze this experiment. We report all data exclusions, all manipulations, and all measures in the study (see^32^). All data and code are publicly available at https://osf.io/ne4r9/.

We tested two simplifications of the intervention implemented in Experiment 1: providing no feedback on the questionnaire and providing only a relevant definition of the personality dimension (see also Fig. 1 for an illustration of the differences in the experimental setup). Participants were randomly assigned to one of four conditions in a 2 (Intervention relevance: boosting vs. control) $\times$ 2 (Intervention type: Definition only vs. Definition + Questionnaire) between-subjects design. In both boosting conditions, participants first received a description of the relevant personality dimension: extraversion–introversion (see Fig. S7). In the questionnaire condition, participants then additionally completed the relevant extraversion inventory (see Fig. S3), but did not receive any feedback. All participants were then asked to identify ads targeted towards their personality. After the ad targeting detection task, participants in both boosting conditions were given feedback on their relative extraversion score (as in Experiment 1, see Figs. 1a, S5, and S6); they then completed the ATI questionnaire and were given feedback on their relative ATI score (see Fig. S6). Because all feedback was provided *after* the detection task, it could not have any effect on the detection task; we included the feedback simply to satisfy participants’ curiosity. For the two control conditions, the position of the extraversion and ATI descriptions (and, in the case of the condition with questionnaire, the corresponding questionnaire) was switched. For the preregistered model’s syntax for Experiment 2, see the Supplemental Information.

638 participants (boosting condition with questionnaire $N=173$, boosting condition without questionnaire $N=130$, control condition with questionnaire $N=164$, control condition without questionnaire $N=171$, randomly allocated on the fly), were recruited from Prolific Academic. All participants provided their informed consent and received £2 for completing the study. No data that allows any identification of participants is reported here, all participants provided informed consent on the data handling. Experiment 2 involved three preregistered prescreening criteria on Prolific, namely, that they had not participated in Experiment 1, its pilot, or a pilot study for Experiment 2.

Consistent with the preregistered exclusion criteria, we excluded participants for the following reasons: 78 participants for non-completion (16 in the boosting condition with questionnaire, 10 in the boosting condition without questionnaire, 29 in the control with questionnaire, 23 in the control without questionnaire), 5 participants for an extraversion percentile of exactly 0.5 (3 in the boosting condition without questionnaire, 2 in the control with questionnaire), 2 participants for giving different responses for the two age questions (1 in the boosting condition with questionnaire, 1 in the control with questionnaire), and 10 participants for failing the comprehension check (3 in the boosting condition with questionnaire, 3 in the boosting condition without questionnaire, 2 in the control with questionnaire, 2 in the control without questionnaire). Our final sample size was thus 544 participants: boosting condition with questionnaire: $N=153$ (i.e., 88% retained); boosting condition without questionnaire: $N=114$ (i.e., 88% retained); control condition with questionnaire: $N=131$ (i.e., 80% retained); and control condition without questionnaire: $N=146$ (i.e., 85% retained). The median age of participants was 29 years (first and third quartiles: $Q_1 = 24$ and $Q_3 = 34$ years).

### Results

**Figure 3:**
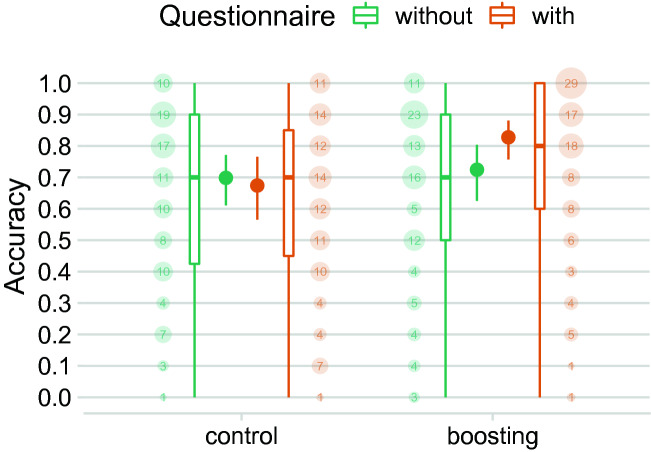
Effects of individual components of the full intervention from Experiment 1 on the ability to accurately detect targeted advertisements (Experiment 2). Participants in the boosting conditions either just read a description of the relevant personality dimension prior to the task (“without questionnaire”; see Fig. 1c), or additionally filled out the short questionnaire from Experiment 1, but without feedback (“with questionnaire”; see Fig. 1d). Point ranges show the Bayesian point estimate and 95% Bayesian credible interval for the probability of correctly detecting a targeted advertisement (based on a mixed-level logistic regression model; see Supplemental Informations for details). In the boxplots, the box shows the the first, second (median), and third quartiles (the 25th, 50th, and 75th percentiles). The lower and upper whiskers extend from the respective end of the box to the largest value no further than 1.5 $\times$ IQR from the box (where IQR is the inter-quartile range, or distance between the first and third quartiles); outliers are not displayed. The area of the dots and their numbers denote the within-condition percentage of participants for each of the 11 possible values for a participant’s proportion of correct decisions (given 10 ads). Figure produced using R version 4.1.0^38^.

The results of Experiment 2 support hypothesis **H2b** (Fig. 3): reflecting on one’s relevant personality dimension—without receiving any relevant feedback—is necessary, but also sufficient to boost people’s ability to identify ads that have been targeted at them. The boosting condition that included the extraversion questionnaire improved participants’ performance by, on average, 10 percentage points (95% CI 2–20) compared to the boosting condition with only the extraversion description, raising mean accuracy from 72% (95% CI 62–80) to 83% (95% CI 76–88); this difference corresponds to a common language effect size of $CL = .62$ (95% CI .52–.71). This positive effect is at odds with hypothesis **H2c**, according to which explicit knowledge of one’s level on the relevant personality dimension is necessary for the intervention to work.

By contrast, participants who only read the extraversion description performed no better than participants who read the unrelated description of the ATI personality dimension ($CL = .52$, 95%: .43–.62); the latter participants correctly identified 70% of the ads (95% CI 61–77). This result is at odds with hypothesis **H2a**, according to which hinting at the strategy used by the advertiser is sufficient for the intervention to work. Importantly, the effectiveness of self-reflection was not generic: performance was boosted only when people reflected on the relevant personality dimension. Participants who read the unrelated description of ATI and then completed the ATI questionnaire correctly identified 67% of the targeted ads (95% CI 57–77)—that is, 15 percentage points (95 CI 7–25) fewer than the participants who reflected on the relevant personality dimension (i.e., extraversion; $CL = .66$, 95%: 58–74).

Additional analyses, detailed in the Supplemental Information (Figs. S13–S15), attest to the robustness of these results. To summarise, the results hold (a) for both extraverts and introverts, (b) different levels of education; moreover, the effect (c) was stronger for extraverts than for introverts, and (d) also emerged when we measured detection performance independently of any response tendency (lenient vs. strict), in terms of the AUC^40^ (based on participants’ confidence in their detection decisions). However, for moderately extraverted participants, we did not observe an effect of filling out the relevant (vs. unrelated) questionnaire (Fig. S13 & S14); for those participants the explicit feedback about their personality seems necessary for improving their detection accuracy (cf. Experiment 1). In summary, Experiment 2 showed that the boosting intervention can improve detection accuracy even without providing explicit feedback, whereas merely describing the relevant personality dimension was insufficient.

## Discussion

Two experiments demonstrated that prompting people to reflect on a targeted personality dimension—by means of a simple intervention—boosts their ability to identify ads aimed at exploiting their personal vulnerabilities. Providing personalized feedback in the full intervention of Experiment 1 resulted in the strongest improvements, which serves as an existence proof of the effectiveness of such interventions. When testing the components of the full intervention in Experiment 2, we found that merely providing a description of the targeted personality dimension did not enhance detection accuracy. In contrast, completing a short personality questionnaire about the targeted personality dimension, but without any feedback, was sufficient to increase accuracy; however, the full intervention with feedback (Experiment 1) yielded larger improvements in accuracy.

These results resonate with the recent finding that simple interventions, such as exposing people to misinformation strategies, can help to inoculate people against such techniques^41,42^. As an instance of boosting interventions—which aim to foster people’s competences—they have the potential to generalise beyond the immediate context in which they were initially deployed^29,43^, for example to the domain of political advertisement. Boosting interventions also have the advantage that they can often be deployed independently of any platform or technology. That is, they do not need to interface with a platform’s information architecture and are therefore not dependent on the platform’s cooperation (in terms of access and maintaining interoperability). Compared with, say, an intervention where advertisements are labelled within the platform’s interface, an intervention targeting people’s competences may therefore prove to be more robust towards constantly changing technology, advertising strategies, and the tech companies’ level of cooperation. Self-reflection tools aimed at helping people increase their awareness of their vulnerabilities to microtargeting could be deployed on independent apps or websites—or even as “analogue” tools (e.g., a checklist on a printed flyer). In the domain of misinformation, gamified implementations of such “inoculation” interventions have so far shown potential to be embraced by large segments of the population^41^. Clearly, such tools would need to cover a range of the most relevant microtargeting dimensions in order to offer effective protection.

Building on our proof-of-principle, future research should reveal the cognitive mechanisms underlying the beneficial effects we found; examine the extent to which an increased detection ability “immunizes” people against the manipulative power of microtargeting (e.g., in terms of how people evaluate and respond to ads; see also^22^); and investigate the extent to which the intervention effects generalise to other personality dimensions and more diverse populations and thus clarify where they are most impactful (e.g., are such self-awareness interventions particularly effective for unfamiliar personality dimensions?). Finally, studying the extent to which the presented boosting interventions generalise to detecting microtargeting in the political domain is a future priority as it is in this domain that the potential of manipulation through microtargeting is most worrisome.

Our findings also raise a question with potentially broader relevance for the goal of transparency in the online world. In our study, merely describing a personality dimension did not suffice to improve people’s ability to detect microtargeting. What does this mean for measures aiming to achieve transparency by merely describing information to users—such as Google’s https://myactivity.google.com or Facebook’s https://facebook.com/your_information? This is an open question, but, clearly, benevolent choice architects should not assume that a mere description and nominal transparency will automatically produce “effective transparency”. Just because something is technically or in legal terms made “transparent” does not yet guarantee that users are able to or willing to engage with the content. And even if they do, they still may not understand what it means for them (e.g., why do I see this ad and how is it trying to influence me?).

To conclude, our results provide a proof-of-principle that is consistent with a long-term vision in which the knowledge asymmetry between platforms and users is reduced and in which the risk of being manipulated into behaviours that serve specific commercial or political interests is curtailed. For this to happen, a mix of regulation and interventions based on insights and evidence from the behavioral sciences will be indispensable. The promising results in the current study underscore that the behavioral sciences in general, and the boosting approach should play a key role in the research and policy endeavor to help citizens regain some of their individual autonomy in the online world^11,30^.

### Ethics declaration

The study was approved by the IRB committee of the Max Planck Institute for Human Development. All experiments followed the IRB guidelines and all participants provided their informed consent before participating in the study. The example image showing people in Fig. 1b is taken from^3^, where it is courtesy of Caiaimage/Paul Bradbury/OJO+/Getty Images.

## Supplementary Information

Supplementary Information.

## Data Availability

All data and code are publicly available at https://osf.io/ne4r9/.
