# Supplementary material for: Boosting people’s ability to detect microtargeted advertising
Source: Sci Rep. 2021 Jul 30;11:15541. doi: 10.1038/s41598-021-94796-z (PMC8324838; doi:10.1038/s41598-021-94796-z)
Supplement: Supplementary file 1 — Supplementary Information. [file 41598_2021_94796_MOESM1_ESM.pdf]

Supplementary Information: Boosting people's ability to detect microtargeted advertising

Philipp Lorenz-Spreen[1*†], Michael Geers[1], Thorsten Pachur[1], Ralph Hertwig[1], Stephan Lewandowsky[2,3], & Stefan M. Herzog[1*]

*contributed equally; †lorenz-spreen@mpib-berlin.mpg.de

Contents

1 Supplemental Information on Methods **2**
1.1 Experiment 1 . . . 2
1.1.1 Personality norms . . . 2
1.1.2 Statistical analysis . . . 5
1.2 Experiment 2 . . . 5
1.2.1 Personality norms . . . 5
1.2.2 Statistical analysis . . . 5

2 Screenshots of Experiments **8**
2.1 Personality questionnaires . . . 8
2.2 Personality feedback screens . . . 10
2.3 Descriptions of personality dimensions . . . 12
2.4 Comprehension check . . . 13
2.5 Stimuli: The 10 ads from (1) . . . 14

3 Additional results **15**
3.1 Experiment 1 . . . 15
3.1.1 Detection performance, boosting intervention, and level of extraversion . . 15
3.1.2 Summary of mixed-level logistic regression model . . . 18
3.2 Experiment 2 . . . 19
3.2.1 Detection performance, boosting intervention, and level of extraversion . . 19
3.2.2 Summary of mixed-level logistic regression model . . . 23

1 Supplemental Information on Methods

1.1 Experiment 1

1.1.1 Personality norms

Questions and distributional information for the raw personality scores were adopted from (2) for extraversion and from (3) for ATI. (2)[1] provide the mean and standard deviation (SD) of the raw scores for each age year between 21 and 60 based on a large Internet study ($N = 132,515$, 91% of participants are from the United States and 9% from Canada; no gender-specific norms were available); we were thus able to provide age-matched feedback for extra-/introversion (for participants aged 18–20 years, we used the norms for age 21 years). For ATI, we used the mean and SD of the sample "S5-full" reported in (3) (i.e., no age- or gender-specific norms were available; this sample is a mix of German and US American Mechanical Turk respondents). To achieve consistency across questionnaires, we presented both questionnaires on a 5-point Likert scale. Because the ATI norm study (3) used a 6-point scale, we rescaled the mean and SD (original norm values $M = 3.61$, $SD = 1.09$; rescaled values $M = 3.09$, $SD = 0.86$). See the screenshots further below for extraversion and ATI questionnaires and sample feedback (Figs. S3 and S4 for questionnaires; Figs. S5 and S6 for feedback and definitions).

Figure S1 shows the distributions of the extraversion and ATI percentiles calculated based on the respective norms. The results show that our female UK participants are somewhat more introverted than their age-matched US counterparts and slightly more technology affine than the ATI population. For the following three reasons, we argue that this difference in the level of extraversion does not pose a problem to the validity of our results and that using local norms (i.e., participants' empirical position in the distribution of raw mean scores in our study, or, in short, in-sample percentiles) is a worse and not better approach.

First, several studies showed that the US and UK populations are similar in terms of extraversion and therefore using a US sample to calculate a UK respondent's extraversion percentile should, in principle, yield similar extraversion percentiles compared with using a UK sample. From this it then follows that the extraversion percentiles we used to give extraversion feedback (Experiment 1) and evaluate the targeting decisions (Experiments 1 and 2) should roughly align with the respondents placement within their overall population (i.e., all UK residents). Here we highlight three empirical patterns to support this claim: (i) The mean and standard deviation of the distributions of extraversion raw scores of US and UK samples are highly similar (see Table 5 in (5)). (ii) Both our US norm sample (2) and a British household panel study (6) show that extraversion decreases slightly from 18 to 40 years. (iii) In both US and UK samples women reported slightly higher extraversion than men (7). In principle, it would be desirable to use UK- and gender-specific extraversion norms. However, even though there are studies reporting British data (e.g., (5; 6; 7)), we have not yet been able to locate a study (or supplemental material or data) that actually reports mean and standard deviations of the raw scores for the British population, which are necessary to compute global percentiles based on an observed raw score.

[1]See `https://web.archive.org/web/20190427124300/http://www.ocf.berkeley.edu/~johnlab/pdfs/BFI%20Comparison%20Samples%20(Ages%2021%20-%2060).doc`.

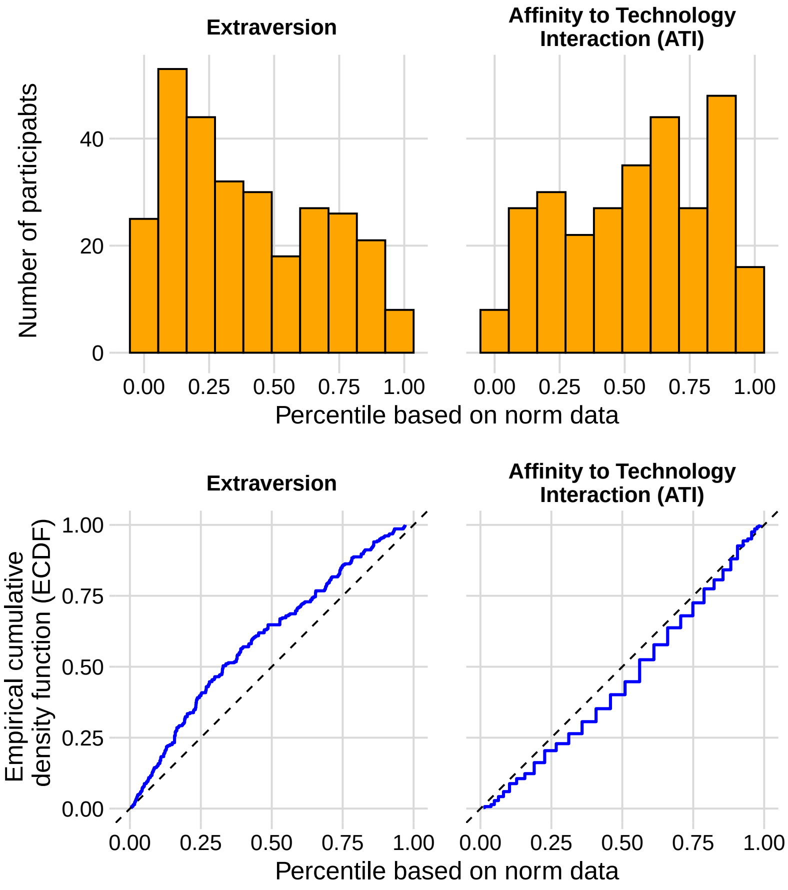

Figure S1: **Distributions of the extraversion and ATI percentiles in Experiment 1**. The top row shows histograms and the bottom row shows empirical cumulative distribution functions (ECDF) of the percentiles for extraversion and ATI, respectively (based on the respective norm data). If participants in Experiment 1 were to completely align with the norm data (2; 3), then the histograms would be uniformly distributed and the ECDFs would lie on the main diagonal. Results show that our female UK participants are somewhat more introverted than their age-matched US counterparts and slightly more technology affine than the ATI population. See text for a discussion of these results. Figure produced using R version 4.1.0 (4).

Second, since a person's level of extraversion is defined relative to a global population (e.g., all UK residents) and not relative to whatever study sample they might end up in, there are conceptual reasons why local norms (i.e., in-sample percentiles) are not per se more relevant than global norms (based on an estimate of the population's mean and standard deviation of the raw score of the personality scale)—and potentially even detrimental for the goals of our study. Consider the extreme case where a study recruits a subpopulation of people known to be

very introverted. Then parts of the more extraverted half of the resulting sample of participants would still be less extraverted than some of the more introverted people in the global population. In other words, even though these participants will have higher in-sample extraversion percentiles compared to their full-population percentiles (e.g., derived based on data, such as reported in (2)), there is no reason to expect them to now respond more to extraverted ads just because they are part of a study that oversampled introverted people. Because our study uses validated stimuli from (1), we aimed to recruit from a population as close as possible to the one microtargeted in that study (i.e., female UK residents ages 18–40; see Methods in the main text for more details). This subpopulation, and the resulting two samples in our study, may not—but also do not need to be—representative of the population of all UK residents in terms of extraversion. As argued above, our goal is assess a participant's level of extraversion relative to the global population and to achieve this, we should use estimates for the mean and standard deviation for that global population.

Third, there are also empirical reasons why using global norms in our study seems preferable to using local norms. Let us assume now that, for whatever reason, in-sample percentiles were actually more representative or relevant for the participants in our two experiments and using those in-sample percentiles would allow us to more truthfully classify participants as extraverted or introverted. If so, then one would expect that re-evaluating participants' targeting decisions according to their in-sample personality category (i.e, calculating their in-sample extraversion percentile, categorizing them as extra- or introverted, and then re-scoring the accuracy of their targeting decisions) should not deteriate participants' apparent detection performance. If anything, one could expect their apparent performance to improve if in-sample extraversion percentiles indeed were more valid (assuming that people, on average, or more likely than not to correctly assess themselves as extra- or introverted.). To empirically assess this conjecture, we conducted a re-analysis of our data. We only included participants from the three control conditions (across both experiments); we did not include participants from the boosting conditions to avoid confounding our analysis with the extraversion feedback (Experiment 1) or exposure to the extraversion definition or questionnaire (Experiment 2). From the total of 422 control participants, 73 (17%) participants previously categorized as introverted would now be re-categorized as extraverted; given that each participants' in-sample percentile is higher than their global percentile (see Figures S1 & S2), none of the participants previously categorized as extraverted changed their assignment. After switching the personality category of a participant, the new accuracy (i.e., proportion correct detection decisions) will, by necessity, be the complement of the original accuracy because now the ground truth of an ad for that participant has switched, while the detection decisions themselves have not (e.g., if accuracy previously was 40%, it will now be 60%; if it previously was 80%, it will now be 20%). Results showed that from the 73 control participants that were re-classified as extraverted, only 6 (8%) improved their accuracy. Thirteen (18%) participants with an original accuracy of 50% stayed at 50%—by necessity because the complement of 50% is again 50%. Markedly, fifty-four (74%) participant had a lower accuracy when using their in-sample extraversion percentile. Across all 73 participants, the median participant's accuracy dropped by 40 percentage points. In sum, this re-analysis suggests that the in-sample extraversion percentiles are not better aligned with participants' personality because control participants performed better if they were scored using their global extraversion percentiles based on (2). This then suggests to us that using the global percentiles, as we did

in this study, is the preferable approach.

1.1.2 Statistical analysis

We used a Bayesian mixed-level logistic regression model implemented in the R package *brms* (8; 9) and its default, vague priors (see code for exact specifications). The preregistered model's syntax is

```
correct ~ 1 + condition + (1 | id) + (1 + condition | stimuli)
```

where `correct` is `1` for correct and `0` for incorrect classification decisions, `condition` is a deviation-coded factor variable for the boosting vs. control condition, `id` is a unique identifier for participants, and `stimuli` is a unique identifier for ads. Note that `(1 + condition | stimuli)` allows the treatment effect to differ in size by ad. Four Markov chain Monte Carlo (MCMC) chains, each with 8,000 samples, were run; the first 4,000 samples were discarded as warm-up. The MCMC diagnostics indicated good convergence (see section 3.1.2 below).

Posterior distributions were summarized using the median (point estimate) and 95% credible interval (uncertainty interval). Based on the model parameters (see section 3.1.2 below for a summary table), we derived posterior distributions for several key statistics of interest: (a) the probability of a correct detection decision in both conditions, (b) the percentage point difference, and (c) effect sizes between the two conditions.

We express effect sizes using the "common language effect size" (CL; 10), which indicates the probability that a randomly selected participant from one condition has a higher value than a randomly selected participant from another condition; a value of 0.5 implies no difference and 1 would imply perfect separation between conditions. CL is well suited to compare conditions in a mixed-level logistic regression model because—unlike the commonly used measures of effect size based on standardized mean differences—CL is invariant to monotonical transformations. That is, its value does not depend on the arbitrary decision about whether to look at the results in log-odds or probability space. We derive the posterior distribution of a CL-comparison based on the model's posterior distributions for the participant-population mean and standard deviation in each condition (setting the item effects to zero, that is, considering the average item).

1.2 Experiment 2

1.2.1 Personality norms

Experiment 2 exactly followed Experiment 1 (see section 1.1.1). Figure S2 shows the distributions of the extraversion and ATI percentiles calculated based on the respective norms. The results show that our female UK participants are somewhat more introverted than their age-matched US counterparts and similarly technology affine as the ATI population. See section 1.1.1 for an in-depth discussion of why this difference in the level of extraversion does not pose a problem to the validity of our results.

1.2.2 Statistical analysis

The preregistered model's syntax is

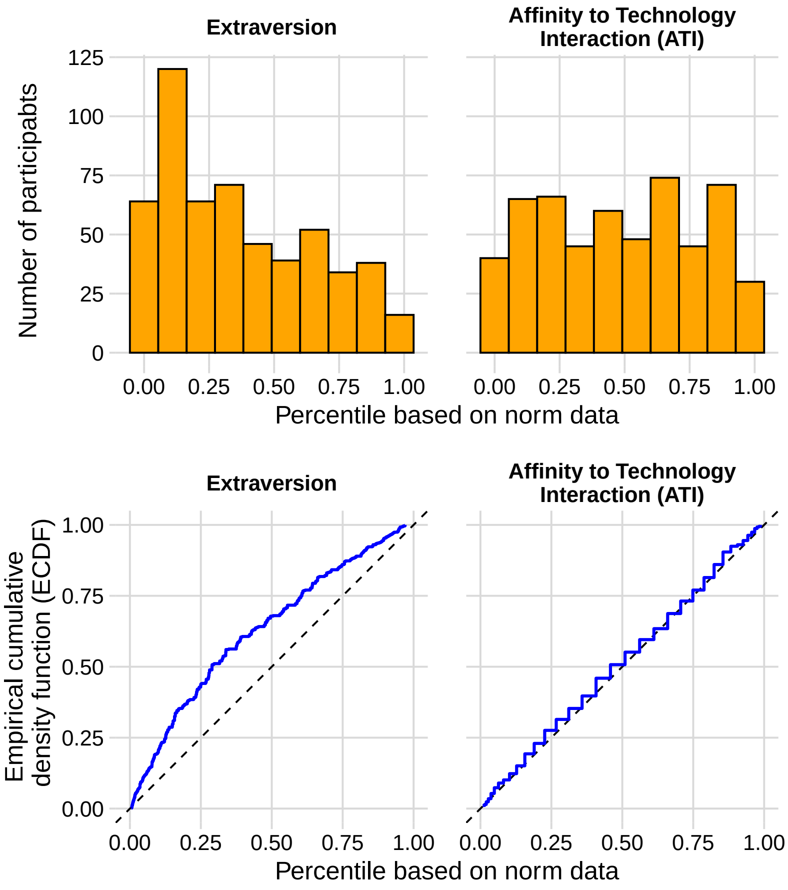

Figure S2: **Distributions of the extraversion and ATI percentiles in Experiment 2**. The top row shows histograms and the bottom row shows empirical cumulative distribution functions (ECDF) of the percentiles for extraversion and ATI, respectively (based on the respective norm data). If participants in Experiment 2 were to completely align with the norm data (2; 3), then the histograms would be uniformly distributed and the ECDFs would lie on the main diagonal. Results show that our female UK participants are somewhat more introverted than their age-matched US counterparts and similarly technology affine as the ATI population. See text for a discussion of these results. Figure produced using R version 4.1.0 (4).

```
correct ~ 1 + relevance * questionnaire + (1 | id)
+ (1 + relevance * questionnaire | stimuli)
```

where `correct` is `1` for correct and `0` for incorrect classification decisions, `relevance` is a deviation-coded factor variable for the boosting vs. control conditions (i.e., relevant vs. unrelated personality dimension, respectively), `questionnaire` is a deviation-coded factor variable indicating whether or not participants were administered a questionnaire, `id` is a unique identifier

for participants, and `stimuli` is a unique identifier for ads. `relevance * questionnaire` indicates that the model includes the two main effects as well as the interaction `relevance : questionnaire`. Note that

```
(1 + relevance * questionnaire | stimuli)
```

allows the treatment effects (i.e., two main effects and their interaction) to differ in size by ad. Four MCMC chains, each with 8,000 samples, were run; the first 4,000 samples were discarded as warm-up. The MCMC diagnostics indicated good convergence (see section 3.2.2 below). Based on the model's parameters (see section 3.2.2 below for a summary table), we derived posterior distributions for several key statistics of interest: (a) the probability of a correct detection decision in each condition, (b) percentage point differences, and (c) effect sizes between conditions. For more information on the analysis approach, see section 1.1.2 above.

2 Screenshots of Experiments

2.1 Personality questionnaires

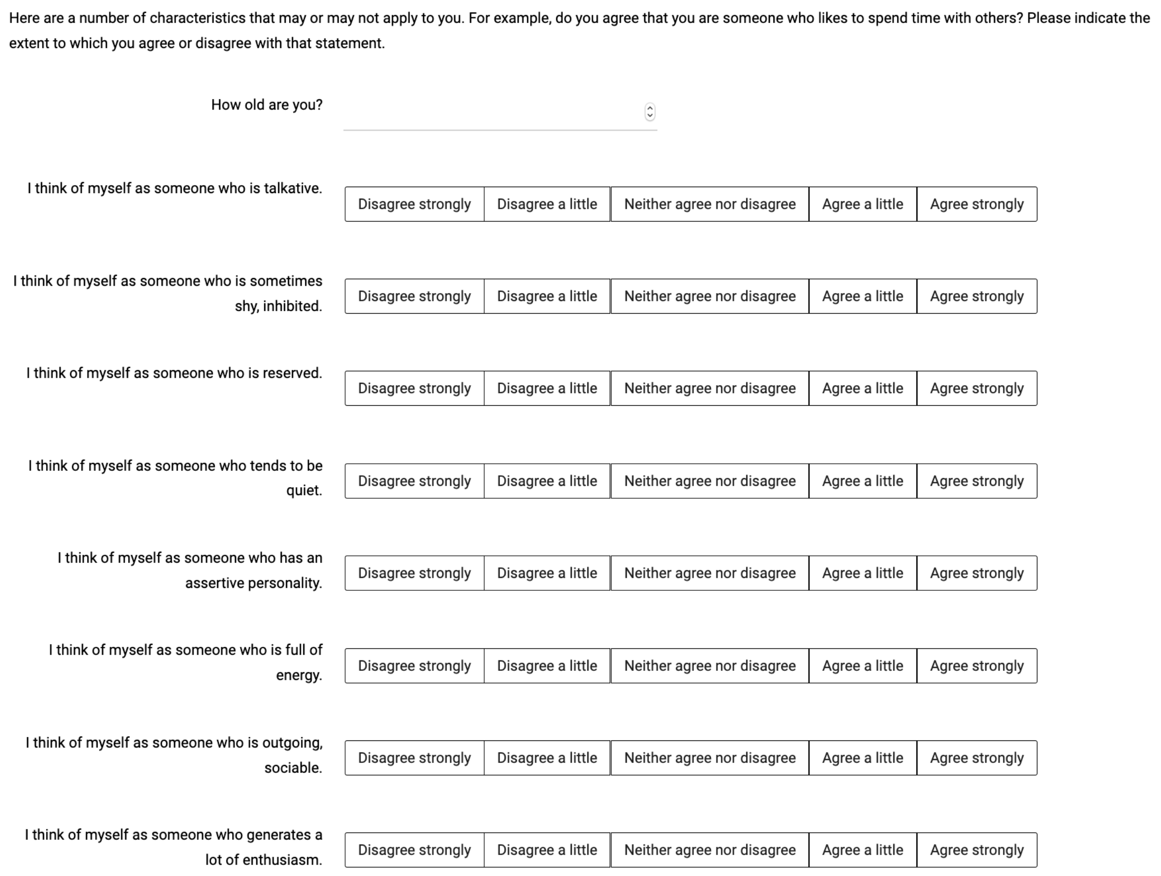
Here are a number of characteristics that may or may not apply to you. For example, do you agree that you are someone who likes to spend time with others? Please indicate the extent to which you agree or disagree with that statement.

How old are you?

I think of myself as someone who is talkative.	Disagree strongly	Disagree a little	Neither agree nor disagree	Agree a little	Agree strongly
I think of myself as someone who is sometimes shy, inhibited.	Disagree strongly	Disagree a little	Neither agree nor disagree	Agree a little	Agree strongly
I think of myself as someone who is reserved.	Disagree strongly	Disagree a little	Neither agree nor disagree	Agree a little	Agree strongly
I think of myself as someone who tends to be quiet.	Disagree strongly	Disagree a little	Neither agree nor disagree	Agree a little	Agree strongly
I think of myself as someone who has an assertive personality.	Disagree strongly	Disagree a little	Neither agree nor disagree	Agree a little	Agree strongly
I think of myself as someone who is full of energy.	Disagree strongly	Disagree a little	Neither agree nor disagree	Agree a little	Agree strongly
I think of myself as someone who is outgoing, sociable.	Disagree strongly	Disagree a little	Neither agree nor disagree	Agree a little	Agree strongly
I think of myself as someone who generates a lot of enthusiasm.	Disagree strongly	Disagree a little	Neither agree nor disagree	Agree a little	Agree strongly

Figure S3: **Extraversion personality questionnaire** used in Experiments 1 and 2. These 8 items are a subset of the 44-items extraversion scale (2)

.

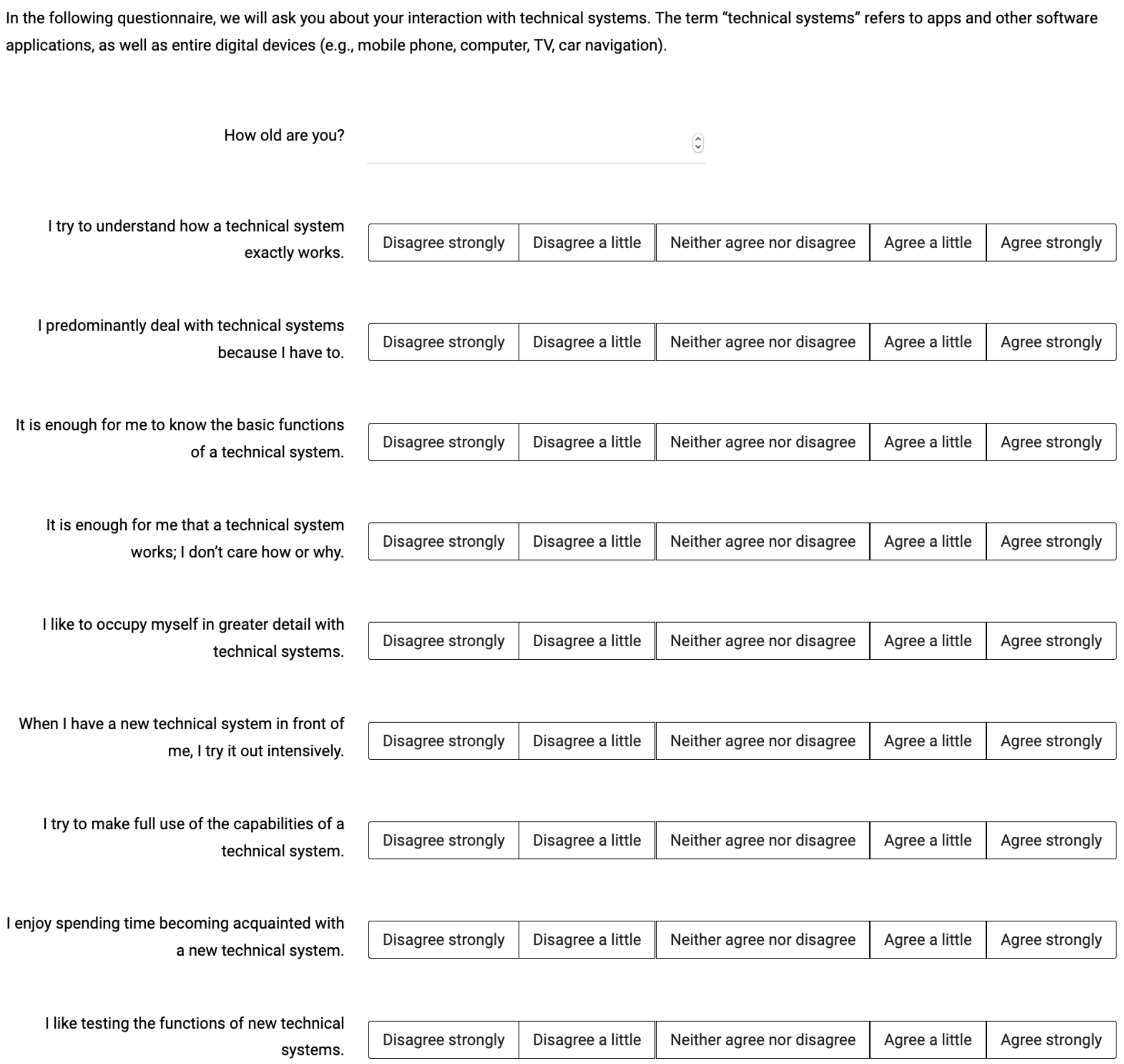
In the following questionnaire, we will ask you about your interaction with technical systems. The term “technical systems” refers to apps and other software applications, as well as entire digital devices (e.g., mobile phone, computer, TV, car navigation).

How old are you?

I try to understand how a technical system exactly works.	Disagree strongly	Disagree a little	Neither agree nor disagree	Agree a little	Agree strongly
I predominantly deal with technical systems because I have to.	Disagree strongly	Disagree a little	Neither agree nor disagree	Agree a little	Agree strongly
It is enough for me to know the basic functions of a technical system.	Disagree strongly	Disagree a little	Neither agree nor disagree	Agree a little	Agree strongly
It is enough for me that a technical system works; I don’t care how or why.	Disagree strongly	Disagree a little	Neither agree nor disagree	Agree a little	Agree strongly
I like to occupy myself in greater detail with technical systems.	Disagree strongly	Disagree a little	Neither agree nor disagree	Agree a little	Agree strongly
When I have a new technical system in front of me, I try it out intensively.	Disagree strongly	Disagree a little	Neither agree nor disagree	Agree a little	Agree strongly
I try to make full use of the capabilities of a technical system.	Disagree strongly	Disagree a little	Neither agree nor disagree	Agree a little	Agree strongly
I enjoy spending time becoming acquainted with a new technical system.	Disagree strongly	Disagree a little	Neither agree nor disagree	Agree a little	Agree strongly
I like testing the functions of new technical systems.	Disagree strongly	Disagree a little	Neither agree nor disagree	Agree a little	Agree strongly

Figure S4: **Affinity for Technology Interaction (ATI) questionnaire** used in Experiments 1 and 2. Items are taken from Franke et al.(3).

2.2 Personality feedback screens

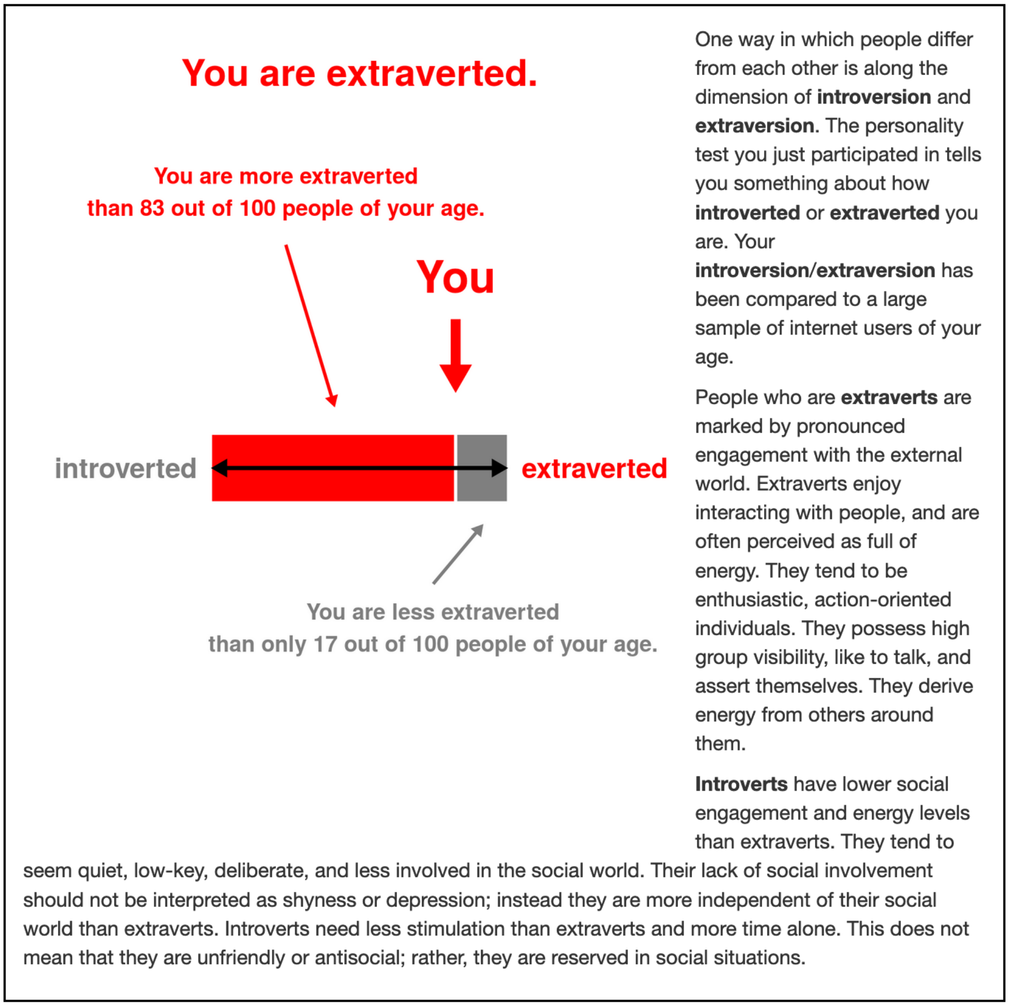

Figure S5: **Personality feedback and description used in the boosting condition in Experiment 1** (i.e., the relevant personality dimension: extraversion). This screenshot is an example for a participant classified as extravert; for participants classified as introverts, the the feedback is reframed in terms of intraversion (i.e., the title reads "You are introverted" and the text below reads "You are more introverted than [XX] out of 100 people of your age" and "You are less introverted than [100 – XX] out of 100 people of your age", where [XX] is the respective percentile). This definition of extraversion is adapted from Wikipedia (`https://web.archive.org/web/20190801042657/https://en.wikipedia.org/wiki/Extraversion_and_introversion`.

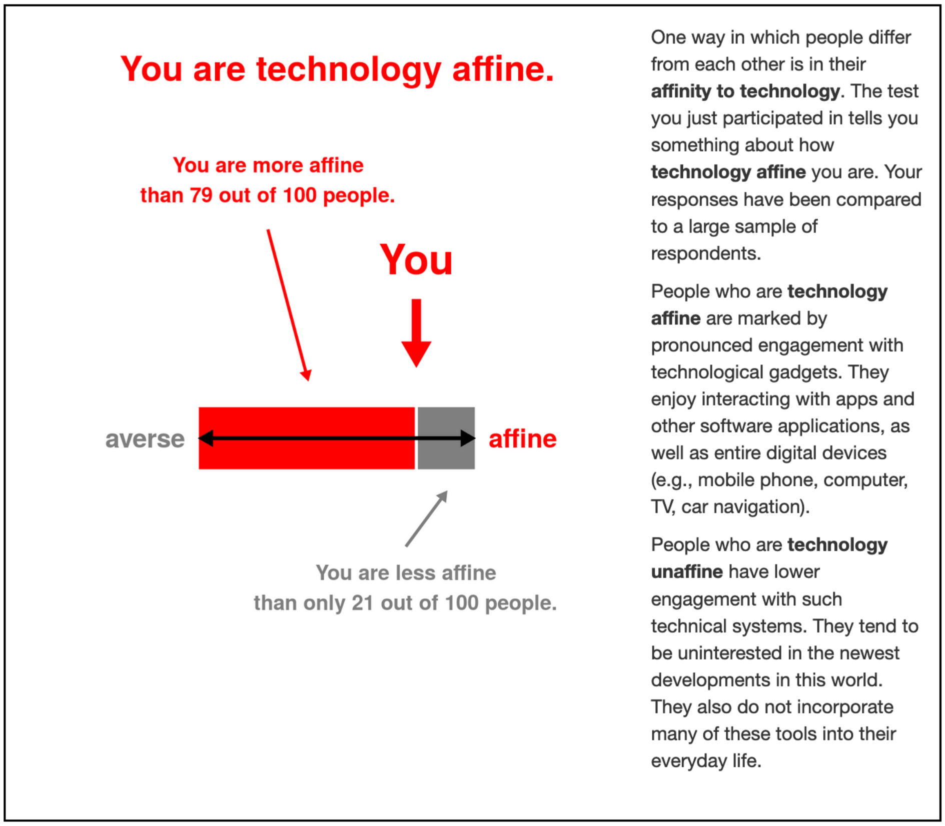

Figure S6: **Personality feedback and description used in the control condition in Experiment 1** (i.e., the irrelevant personality dimension: Affinity for Technology, ATI (3)). This screenshot is an example for a participant classified as technology affine; for participants classified as not technology affine, the the feedback is reframed in terms of technology aversion (i.e., the title reads "You are technology averse" and the text below reads "You are more averse than [XX] out of 100 people" and "You are less averse than [100 – XX] out of 100 people", where [XX] is the respective percentile).

2.3 Descriptions of personality dimensions

Extraversion and introversion

One way in which people differ from each other is along the dimension of **introversion** and **extraversion**.

People who are **extraverts** are marked by pronounced engagement with the external world. Extraverts enjoy interacting with people, and are often perceived as full of energy. They tend to be enthusiastic, action-oriented individuals. They possess high group visibility, like to talk, and assert themselves. They derive energy from others around them.

Introverts have lower social engagement and energy levels than extraverts. They tend to seem quiet, low-key, deliberate, and less involved in the social world. Their lack of social involvement should not be interpreted as shyness or depression; instead they are more independent of their social world than extraverts. Introverts need less stimulation than extraverts and more time alone. This does not mean that they are unfriendly or antisocial; rather, they are reserved in social situations.

Please wait for one minute and read through the above information. Then continue here:

Go on

Figure S7: **Description of the extraversion personality dimension**, used in the boosting condition in Experiment 2. This definition of extraversion is adapted from Wikipedia (https://web.archive.org/web/20190801042657/https://en.wikipedia.org/wiki/Extraversion_and_introversion).

Affinity for technology interaction

One way in which people differ from each other is in their **affinity to technology**.

People who are **technology affine** are marked by pronounced engagement with the technological gadgets. They enjoy interacting with apps and other software applications, as well as entire digital devices (e.g., mobile phone, computer, TV, car navigation).

People who are **technology unaffine** have lower engagement with such technical systems. They tend to be uninterested in the newest developments in this world. They also do not incorporate many of these tools into their everyday life.

Please wait for one minute and read through the above information. Then continue here:

Go on

Figure S8: **Description of the Affinity for Technology scale (ATI)** used in the control condition in Experiment 2. This definition is taken from Franke et al. (3).

2.4 Comprehension check

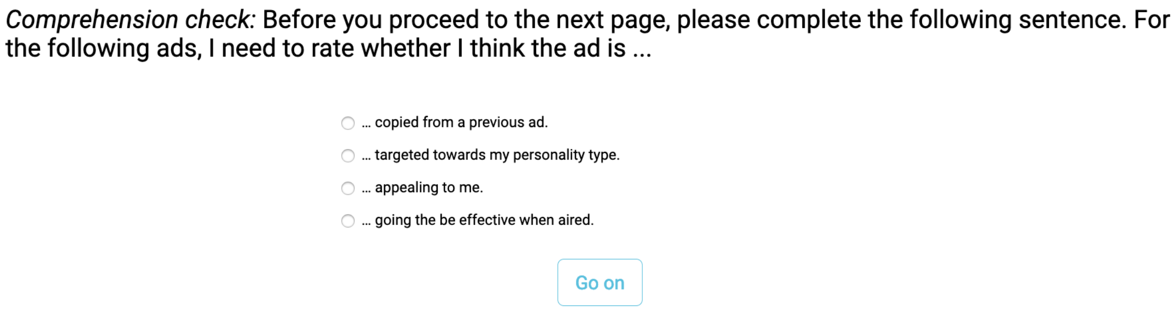
Comprehension check: Before you proceed to the next page, please complete the following sentence. For the following ads, I need to rate whether I think the ad is ...

- ... copied from a previous ad.
- ... targeted towards my personality type.
- ... appealing to me.
- ... going the be effective when aired.

Go on

Figure S9: **Comprehension check used in Experiments 1 and 2 prior to starting the detection task.** If a participant did not choose the correct answer (“targeted towards my personality type”), the questions was shown again up to two more times, alongside the note ”The last answer was not correct, please try again:” (i.e., a total maximum of three attempts). The response options were sorted differently after each incorrect response. Only participants who passed the comprehension check within three attempts were included in the analysis (see 1.1 in the main text and the preregistrations).

2.5 Stimuli: The 10 ads from (1)

Image accessible here: http://www.gettyimages.co.uk/license/137548001<br>Text: Beauty doesn't have to shout	Image accessible here: http://www.gettyimages.ca/license/476806075<br>Text: Dance like no one's watching (but they totally are)
Image accessible here: http://www.gettyimages.co.uk/license/488603139<br>Text: Uncover your natural beauty	Image accessible here: http://www.gettyimages.co.uk/license/476996977<br>Text: Bring out your best features and bring on the night
Image accessible here: http://www.gettyimages.ca/license/78767137<br>Text: Indulge your natural beauty	Image accessible here: http://www.gettyimages.co.uk/license/130899617<br>Text: Love the spotlight and feel the moment
Image accessible here: http://www.gettyimages.co.uk/license/496839201<br>Text: Find some time to feel yourself	Image accessible here: http://www.gettyimages.co.uk/license/152415201<br>Text: Bold characters feel unique
Image accessible here: http://www.gettyimages.co.uk/license/184934575<br>Text: Beauty isn't always about being on show	Image accessible here: http://www.gettyimages.co.uk/license/130406597<br>Text: Love the spotlight

Table S1: **Stimuli: The 10 ads used in Experiments 1 and 2.** The ads in the left column are tailored to extraverts and the ads in the right column to introverts. Images and text were adopted from (1)

3 Additional results

3.1 Experiment 1

3.1.1 Detection performance, boosting intervention, and level of extraversion

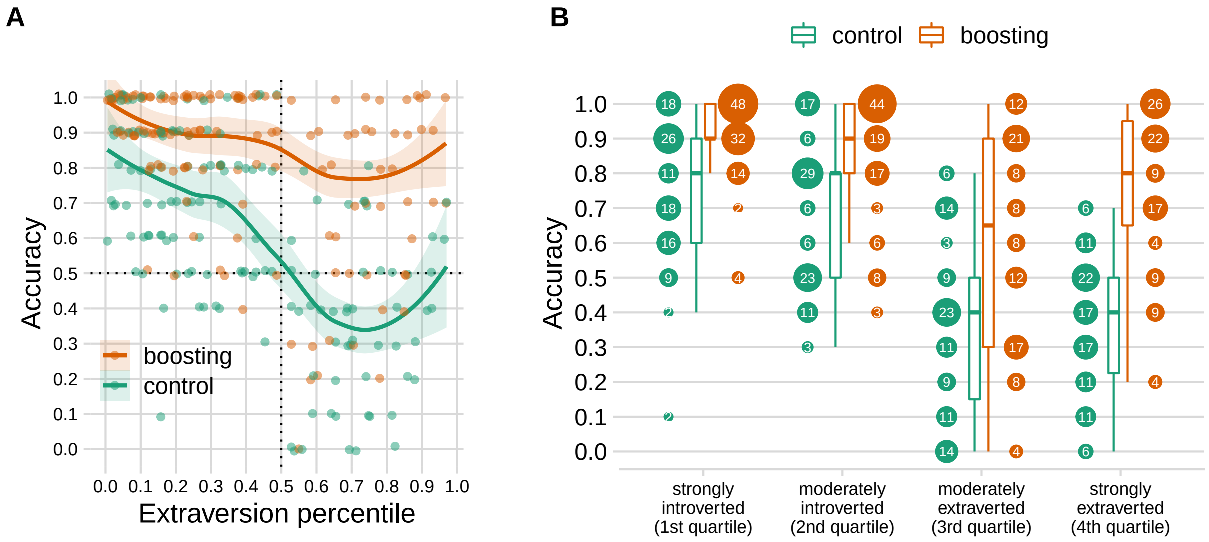

Figure S10: **Detection performance (in terms of proportion of correct decisions), boosting intervention, and level of extraversion (Experiment 1)**. **A** Scatterplot of participants' accuracy (i.e., proportion correct decisions; y-axis) and their extraversion percentile (from 0 most introverted to 1 most extraverted; x-axis) for boosting vs. control group (color coded). Dots are slightly jittered vertically to avoid overplotting. Curves and confidence bands show robust LOESS curves (locally estimated scatterplot smoothing using re-descending M estimator with Tukey's biweight function) and their 95% confidence band. **B** Detection accuracy by extraversion quartiles (x-axis) for boosting vs. control group (color coded). In the boxplots, the box shows the the first, second (median), and third quartiles (the 25th, 50th, and 75th percentiles). The lower and upper whiskers extend from the respective end of the box to the largest value no further than 1.5 × IQR from the box (where IQR is the inter-quartile range, or distance between the first and third quartiles); outliers are not displayed. The area of the dots and their numbers denote the within-quartile-and-condition percentage of participants for each of the 11 possible values for a participant's value of proportion of correct decisions (given the 10 ads). Figure produced using R version 4.1.0 (4).

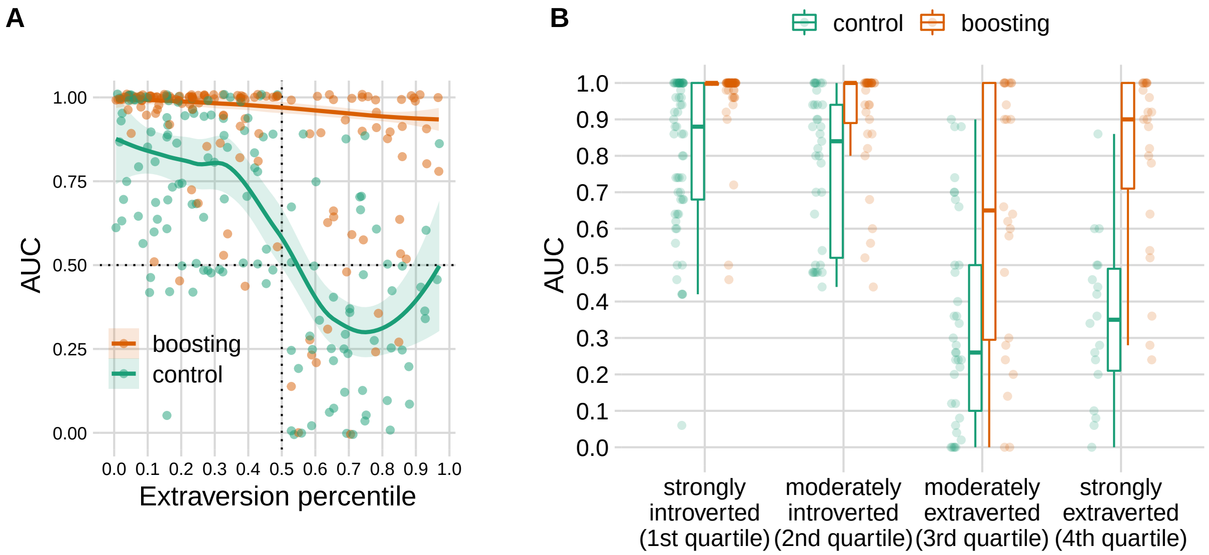

Figure S11: **Detection performance (in terms of the area under the Receiver Operating Characteristics curve, AUC, based on participants' confidence rating), boosting intervention, and level of extraversion (Experiment 1)**. Detection accuracy is quantified using the AUC based on participants' confidence rating, using the trapezoid method (i.e., no kernel- or model-based smoothing; 11). In particular, this calculation uses a participant's confidence that the ad is targeted towards them (implied by the participant's binary categorization decision and corresponding rating about how confident the respondent is in the correctness of her decision). An AUC value can be interpreted as the probability that a participant's confidence (in the sense described above) is higher for a randomly selected ad that actually targets this participant compared to a randomly selected ad that does not actually target this participant. **A** Scatterplot of participants' detection performance (i.e., AUC; y-axis) and their extraversion percentile (from 0 most introverted to 1 most extraverted; x-axis) for boosting vs. control group (color coded). Dots are slightly jittered vertically to avoid overplotting. Curves and confidence bands show robust LOESS curves (locally estimated scatterplot smoothing using re-descending M estimator with Tukey's biweight function) and their 95% confidence band. **B** Detection performance (i.e., AUC; y-axis) by extraversion quartiles (x-axis) for boosting vs. control group (color coded). Dots show individual participants (jittered horizontally to avoid overplotting). In the boxplots, the box shows the the first, second (median), and third quartiles (the 25th, 50th, and 75th percentiles). The lower and upper whiskers extend from the respective end of the box to the largest value no further than 1.5 × IQR from the box (where IQR is the inter-quartile range, or distance between the first and third quartiles); outliers are not displayed. Figure produced using R version 4.1.0 (4).

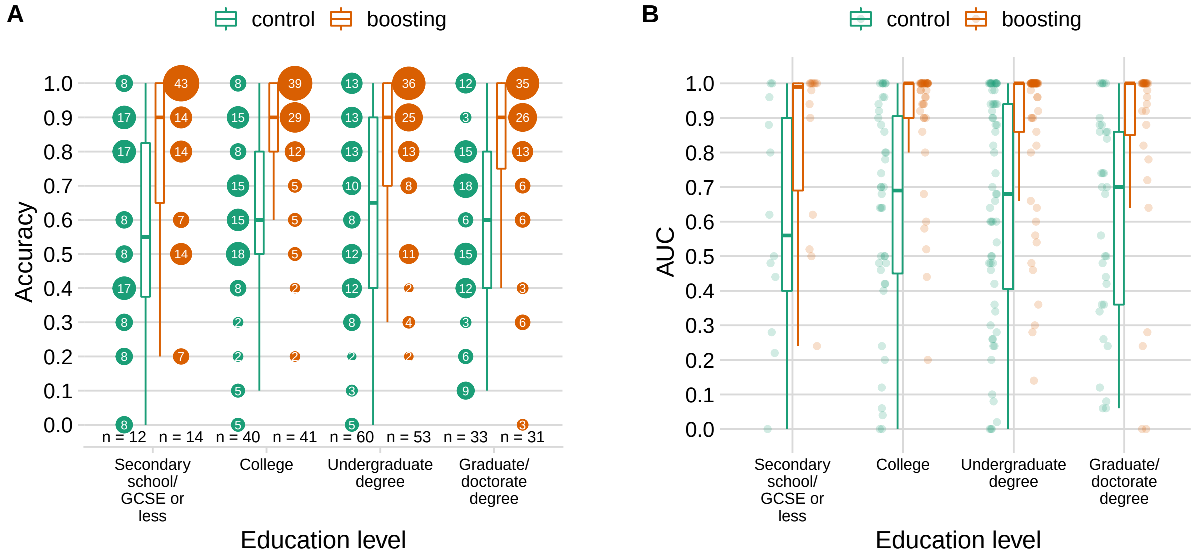

Figure S12: **Detection performance, boosting intervention, and education (Experiment 1).** **A** Detection accuracy (i.e., proportion correct decisions; y-axis) by education (x-axis) for boosting vs. control group (color coded). The area of the dots and their numbers denote the within-education-and-condition percentage of participants for each of the 11 possible values for a participant's value of proportion of correct decisions (given the 10 ads). n denotes the number of participants for each combination of education level and condition. **B** Detection performance in terms of AUC (y-axis); see Fig. S13 for more details on AUC. Dots show individual participants (jittered horizontally to avoid overplotting). In the boxplots, the box shows the the first, second (median), and third quartiles (the 25th, 50th, and 75th percentiles). The lower and upper whiskers extend from the respective end of the box to the largest value no further than 1.5 × IQR from the box (where IQR is the inter-quartile range, or distance between the first and third quartiles); outliers are not displayed. Figure produced using R version 4.1.0 (4).

3.1.2 Summary of mixed-level logistic regression model

The text below shows the model summary of the *brms* Bayesian mixed-level logistic regression model (8; 9) reported for Experiment 1. See section 1.1.2 above for more information on the coding of the variables. `Estimate` shows the median and `l-95%` and `u-95%` show the 95% posterior credibility interval (i.e., the 2.5% and 97.5% percentile, respectively) of the respective marginal posterior distribution. For more details see the R help file `?brms::summary.brmsfit`[2]

```
 Family: bernoulli
  Links: mu = logit
Formula: dec_correct ~ 1 + condition + (1 | id) + (1 + condition | stimuli)
   Data: tbl_targeting_1 (Number of observations: 2840)
Samples: 4 chains, each with iter = 8000; warmup = 4000; thin = 1;
         total post-warmup samples = 16000

Group-Level Effects:
~id (Number of levels: 284)
              Estimate Est.Error l-95% CI u-95% CI Rhat Bulk_ESS Tail_ESS
sd(Intercept)     1.52      0.11     1.32     1.75 1.00     6358    10048

~stimuli (Number of levels: 10)
                          Estimate Est.Error l-95% CI u-95% CI Rhat Bulk_ESS
sd(Intercept)                 0.48      0.14     0.28     0.92 1.00     5852
sd(condition1)                0.20      0.16     0.01     0.63 1.00     6142
cor(Intercept,condition1)     0.29      0.55    -0.83     0.95 1.00    18907
                          Tail_ESS
sd(Intercept)                 9267
sd(condition1)                7713
cor(Intercept,condition1)    10651

Population-Level Effects:
           Estimate Est.Error l-95% CI u-95% CI Rhat Bulk_ESS Tail_ESS
Intercept      1.38      0.20     0.97     1.79 1.00     5350     8842
condition1     1.62      0.24     1.16     2.10 1.00     6967    10456

Samples were drawn using sampling(NUTS). For each parameter, Bulk_ESS
and Tail_ESS are effective sample size measures, and Rhat is the potential
scale reduction factor on split chains (at convergence, Rhat = 1).
```

[2]E.g., at `https://rdrr.io/cran/brms/man/summary.brmsfit.html`.

3.2 Experiment 2

3.2.1 Detection performance, boosting intervention, and level of extraversion

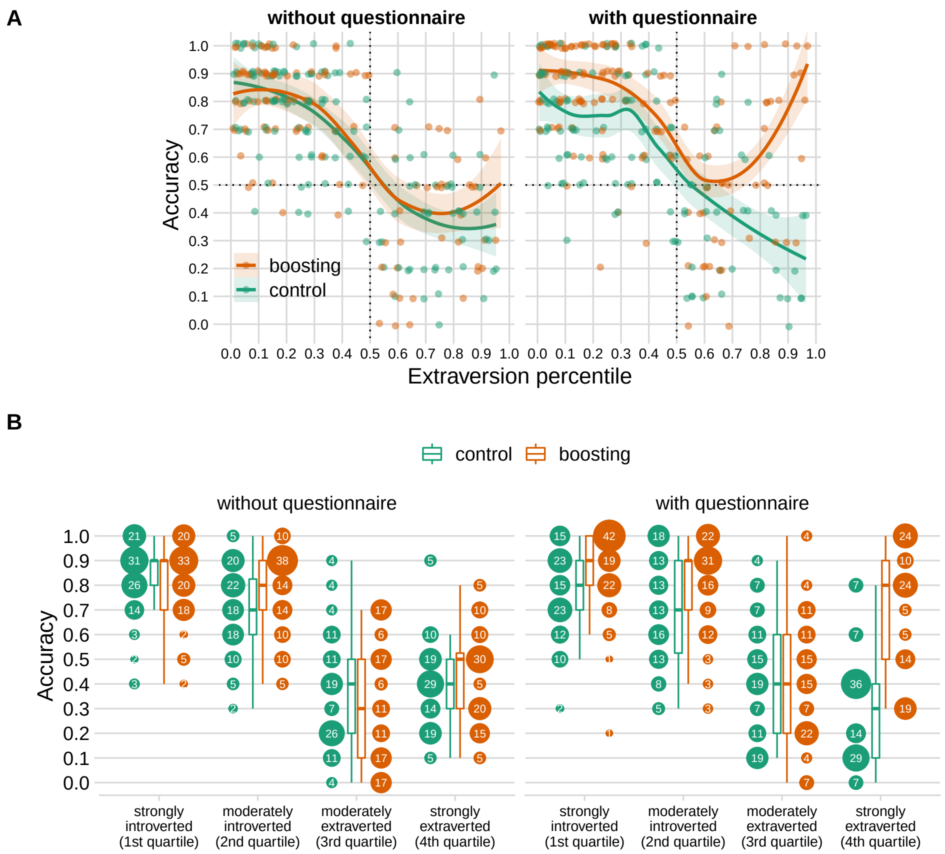

Figure S13: **Detection performance, boosting intervention, and level of extraversion (Experiment 2)**. **A** Scatterplot of participants' accuracy (i.e., proportion correct decisions; y-axis) and their extraversion percentile (from 0 most introverted to 1 most extraverted; x-axis) for boosting vs. control group (color coded) and without and with questionnaire (left & right subplot, respectively). Dots are slightly jittered vertically to avoid overplotting. Curves and confidence bands show robust LOESS curves (locally estimated scatterplot smoothing using re-descending M estimator with Tukey's biweight function) and their 95% confidence band. **B** Detection performance by extraversion quartiles (x-axis) for boosting vs. control group (color coded) and without and with questionnaire (left & right subplot, respectively). In the boxplots, the box shows the the first, second (median), and third quartiles (the 25th, 50th, and 75th percentiles). The lower and upper whiskers extend from the respective end of the box to the largest value no further than 1.5 × IQR from the box (where IQR is the inter-quartile range, or distance between the first and third quartiles); outliers are not displayed. The area of the dots and their numbers denote the within-quartile-and-condition percentage of participants for each of the 11 possible values for a participant's value of proportion of correct decisions (given the 10 ads). Figure produced using R version 4.1.0 (4).

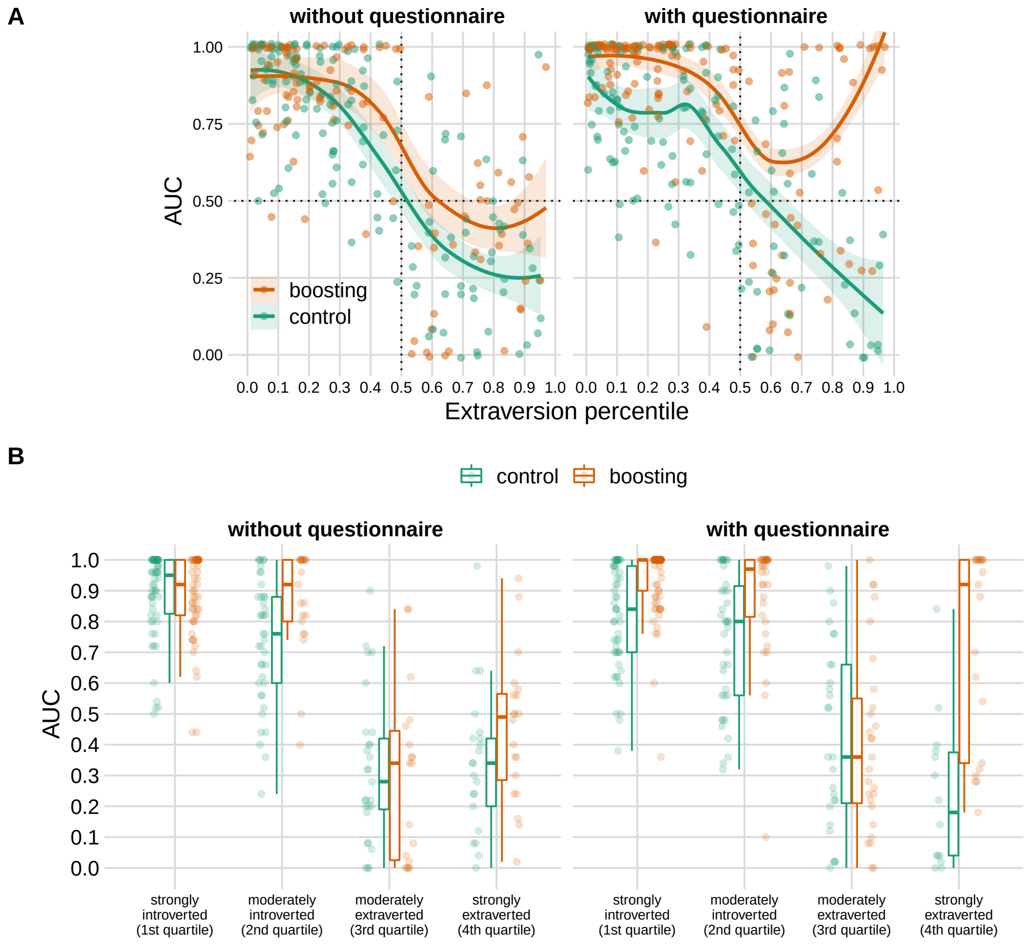

Figure S14: **Detection performance (in terms of the area under the Receiver Operating Characteristics curve, AUC, based on participants' confidence rating), boosting intervention, and level of extraversion (Experiment 2)**. Detection accuracy is quantified using the AUC based on participants' confidence rating, using the trapezoid method (i.e., no kernel- or model-based smoothing; 11). In particular, this calculation uses a participant's confidence that the ad is targeted towards them (implied by the participant's binary categorization decision and corresponding rating about how confident the respondent is in the correctness of her decision). **A** Scatterplot of participants' detection performance (i.e., AUC; y-axis) and their extraversion percentile (from 0 most introverted to 1 most extraverted; x-axis) for boosting vs. control group (color coded) and without and with questionnaire (left & right subplot, respectively). **B** Detection performance (i.e., AUC; y-axis) by extraversion quartiles (x-axis) for boosting vs. control group (color coded) and without and with questionnaire (left & right subplot, respectively). See Fig. S11 for more details on AUC and what the two panels show. Figure produced using R version 4.1.0 (4).

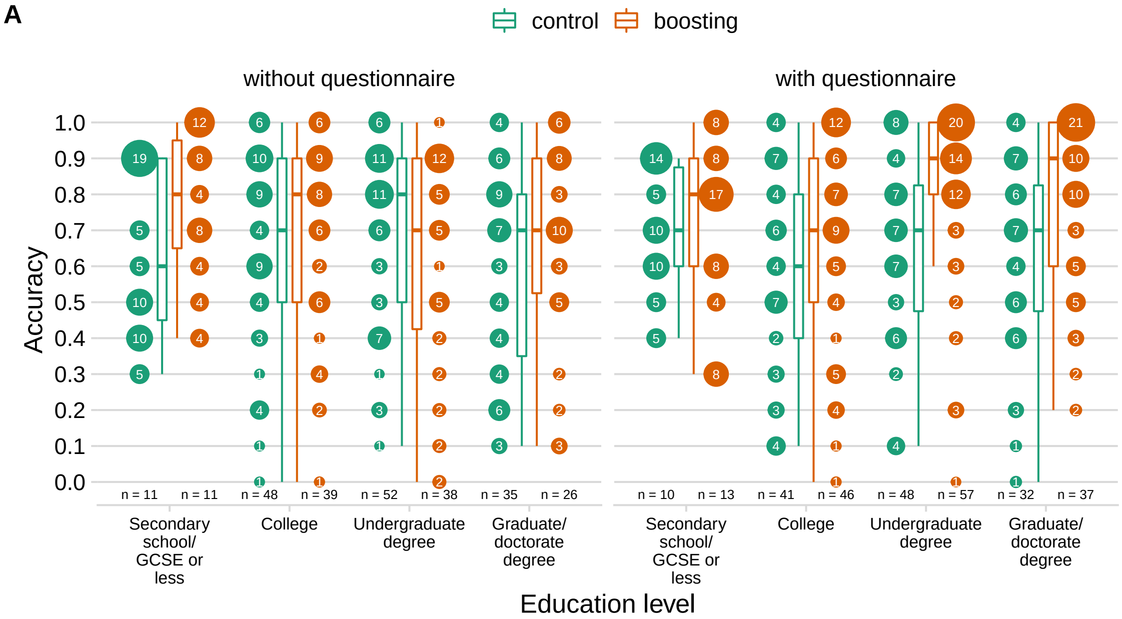

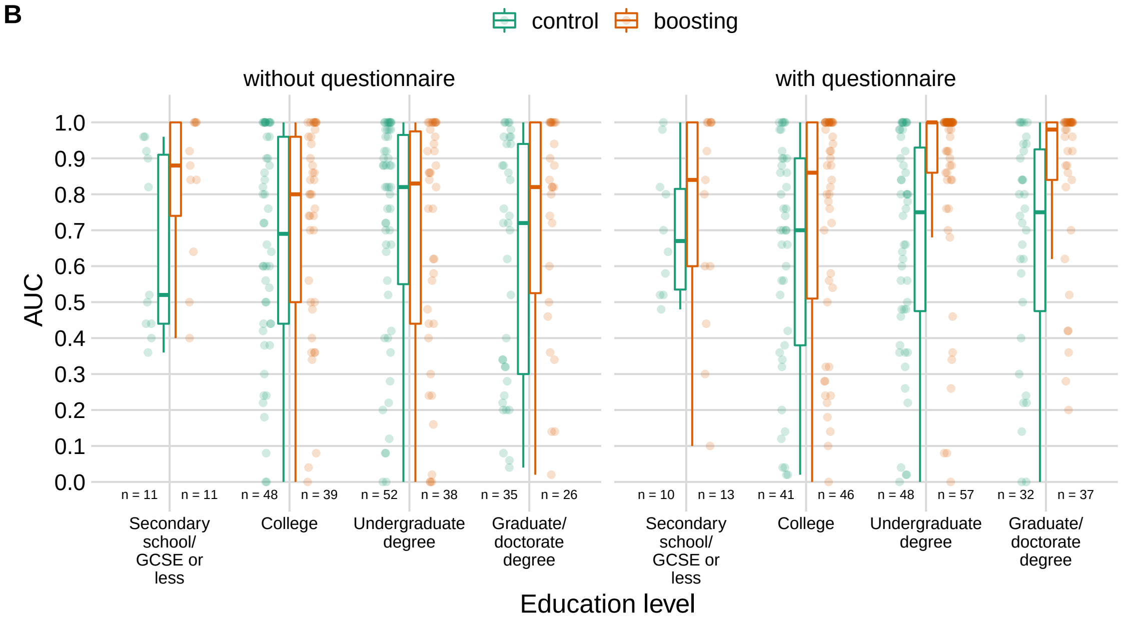

Figure S15: **Detection performance, boosting intervention, and education (Experiment 2)**. **A** Detection accuracy (i.e., proportion correct decisions; y-axis) by education (x-axis) for boosting vs. control group (color coded) and without and with questionnaire (left & right subplot, respectively). **B** Detection performance in terms of AUC (y-axis); see Fig S13 for more details on AUC and Fig S11 for more details on what the two panels show. Figure produced using R version 4.1.0 (4).

3.2.2 Summary of mixed-level logistic regression model

The text below shows the model summary of the *brms* Bayesian mixed-level logistic regression model (8; 9) reported for Experiment 2. See section 1.1.2 above for more information on the coding of the variables. `Estimate` shows the median and `l-95%` and `u-95%` show the 95% posterior credibility interval (i.e., the 2.5% and 97.5% percentile, respectively) of the respective marginal posterior distribution. For more details see the R help file `?brms::summary.brmsfit`[3]

```
 Family: bernoulli
  Links: mu = logit
Formula: dec_correct ~ relevance + questionnaire + (1 | id) + (1 + relevance * questionnaire | stimuli) + relevance:questionnaire
   Data: tbl_targeting_2 (Number of observations: 5440)
Samples: 4 chains, each with iter = 8000; warmup = 4000; thin = 1;
         total post-warmup samples = 16000

Group-Level Effects:
~id (Number of levels: 544)
              Estimate Est.Error l-95% CI u-95% CI Rhat Bulk_ESS Tail_ESS
sd(Intercept)     1.40      0.07     1.27     1.55 1.00     6152    10037

~stimuli (Number of levels: 10)
                                              Estimate Est.Error l-95% CI u-95% CI Rhat Bulk_ESS Tail_ESS
sd(Intercept)                                     0.41      0.12     0.25     0.77 1.00     4342     7611
sd(relevance1)                                    0.07      0.06     0.00     0.28 1.00     9937     6944
sd(questionnaire1)                                0.28      0.13     0.05     0.64 1.00     5235     4153
sd(relevance1:questionnaire1)                     0.58      0.26     0.10     1.32 1.00     5242     4135
cor(Intercept,relevance1)                         0.21      0.50    -0.73     0.88 1.00    20004    10396
cor(Intercept,questionnaire1)                     0.27      0.36    -0.49     0.81 1.00    13771    11535
cor(relevance1,questionnaire1)                    0.01      0.50    -0.80     0.82 1.00     5925    10781
cor(Intercept,relevance1:questionnaire1)         -0.31      0.35    -0.82     0.44 1.00    14273    11177
cor(relevance1,relevance1:questionnaire1)        -0.01      0.51    -0.82     0.80 1.00     6887    10321
cor(questionnaire1,relevance1:questionnaire1)     0.02      0.43    -0.73     0.73 1.00    10467    12535

Population-Level Effects:
                        Estimate Est.Error l-95% CI u-95% CI Rhat Bulk_ESS Tail_ESS
Intercept                   1.03      0.15     0.69     1.34 1.00     3497     5590
relevance1                  0.48      0.15     0.19     0.77 1.00     4468     7879
questionnaire1              0.24      0.17    -0.11     0.59 1.00     5883     8847
relevance1:questionnaire1   0.72      0.35    -0.01     1.42 1.00     5783     8725

Samples were drawn using sampling(NUTS). For each parameter, Bulk_ESS
and Tail_ESS are effective sample size measures, and Rhat is the potential
scale reduction factor on split chains (at convergence, Rhat = 1).
```

[3] E.g., at `https://rdrr.io/cran/brms/man/summary.brmsfit.html`.

References

[1] Matz, S. C., Kosinski, M., Nave, G. & Stillwell, D. J. Psychological targeting as an effective approach to digital mass persuasion. *Proceedings of the National Academy of Sciences* **114**, 12714–12719 (2017).

[2] Srivastava, S., John, O. P., Gosling, S. D. & Potter, J. Development of personality in early and middle adulthood: Set like plaster or persistent change? *Journal of Personality and Social Psychology* **84**, 1041–1053 (2003).

[3] Franke, T., Attig, C. & Wessel, D. A personal resource for technology interaction: Development and validation of the Affinity for Technology Interaction (ATI) scale. *International Journal of Human–Computer Interaction* **35**, 456–467 (2019).

[4] R Core Team. *R: A Language and Environment for Statistical Computing.* R Foundation for Statistical Computing, Vienna, Austria (2021).

[5] Schmitt, D. P., Allik, J., McCrae, R. R. & Benet-Martínez, V. The geographic distribution of big five personality traits: Patterns and profiles of human self-description across 56 nations. *Journal of Cross-Cultural Psychology* **38**, 173–212 (2007).

[6] Donnellan, M. B. & Lucas, R. E. Age differences in the big five across the life span: Evidence from two national samples. *Psychology and Aging* **23**, 558–566 (2008).

[7] Schmitt, D. P., Realo, A., Voracek, M. & Allik, J. Why can't a man be more like a woman? Sex differences in big five personality traits across 55 cultures. *Journal of Personality and Social Psychology* **94**, 168–182 (2008).

[8] Bürkner, P.-C. brms: An R package for Bayesian multilevel models using Stan. *Journal of Statistical Software* **80**, 1–28 (2017).

[9] Bürkner, P.-C. Advanced Bayesian multilevel modeling with the R package brms. *The R Journal* **10**, 395–411 (2018).

[10] Ruscio, J. A probability-based measure of effect size: Robustness to base rates and other factors. *Psychological Methods* **13**, 19–30 (2008).

[11] Fawcett, T. An introduction to ROC analysis. *Pattern Recognition Letters* **27**, 861–874 (2006).
